# POLARIS: Polygenic LD‐adjusted risk score approach for set‐based analysis of GWAS data

**DOI:** 10.1002/gepi.22117

**Published:** 2018-03-12

**Authors:** Emily Baker, Karl Michael Schmidt, Rebecca Sims, Michael C. O'Donovan, Julie Williams, Peter Holmans, Valentina Escott‐Price, with the GERAD Consortium

**Affiliations:** ^1^ Medical Research Council Centre for Neuropsychiatric Genetics and Genomics Institute of Psychological Medicine and Clinical Neurosciences, Cardiff University United Kingdom; ^2^ School of Mathematics Cardiff University United Kingdom

**Keywords:** genetics, GWAS, linkage disequilibrium, MAGMA, polygenic risk score

## Abstract

Polygenic risk scores (PRSs) are a method to summarize the additive trait variance captured by a set of SNPs, and can increase the power of set‐based analyses by leveraging public genome‐wide association study (GWAS) datasets. PRS aims to assess the genetic liability to some phenotype on the basis of polygenic risk for the same or different phenotype estimated from independent data. We propose the application of PRSs as a set‐based method with an additional component of adjustment for linkage disequilibrium (LD), with potential extension of the PRS approach to analyze biologically meaningful SNP sets. We call this method POLARIS: POlygenic Ld‐Adjusted RIsk Score. POLARIS identifies the LD structure of SNPs using spectral decomposition of the SNP correlation matrix and replaces the individuals' SNP allele counts with LD‐adjusted dosages. Using a raw genotype dataset together with SNP effect sizes from a second independent dataset, POLARIS can be used for set‐based analysis. MAGMA is an alternative set‐based approach employing principal component analysis to account for LD between markers in a raw genotype dataset. We used simulations, both with simple constructed and real LD‐structure, to compare the power of these methods. POLARIS shows more power than MAGMA applied to the raw genotype dataset only, but less or comparable power to combined analysis of both datasets. POLARIS has the advantages that it produces a risk score per person per set using all available SNPs, and aims to increase power by leveraging the effect sizes from the discovery set in a self‐contained test of association in the test dataset.

## 1 INTRODUCTION

Polygenic risk scores (PRSs) are now widely used for a variety of purposes in assessing the genetic liability to disorders or more general phenotypes. These include sample stratification, risk prediction, and the detection of relationships between different subphenotypes (see, e.g., Allardyce et al., 2017; Escott‐Price et al., 2015, and Foley et al., 2017, respectively). The PRS method can also be adapted to partition the polygenic risk based on meaningful SNP sets, such as genes or biological pathways, and to determine whether a set of SNPs, weighted with their individual genetic risk effects, is associated at the whole‐genome or set‐specific levels. In contrast to set analysis that aims to analyze the joint association of SNPs with a single phenotype, PRS aims to assess the genetic liability to some phenotype on the basis of the polygenic risk for the same or a different phenotype estimated from independent data.

Set‐based analysis offers an attractive alternative to single SNP analyses, because the combined effect of SNPs within the set may be captured. Single SNP analyses are often underpowered due to the small effect sizes of individual SNPs, set‐based analysis considers the combined effect of all SNPs within the set, which may have a larger combined effect size and hence higher power to detect association than any individual SNP. In addition, gene‐based analysis, a gene‐centered equivalent of set‐based analysis, identifies genes associated with disease rather than a single SNP as a proxy for the gene. In gene‐based analyses, genes are found to be fairly consistently associated with disease across different populations. In contrast, different SNPs in a set in linkage disequilibrium (LD) may be found to be associated with a disease in different samples. Gene‐based analyses also directly provide information for functional analysis (Li, Gui, Kwan, & Sham, 2011). Set‐based analysis can also be employed as a pathway analysis, and applied to sets of SNPs defined by epigenomics for different tissue/cell types.

There are a number of methods to assess the set‐based effect by combining the effects of all SNPs within the set, including Fisher's method (Elston, 1991) for combining *P* values assuming independence between SNPs, Simes's method (Simes, 1986) for finding the smallest adjusted *P*‐value, GATES extended Simes (Li et al., 2011) that incorporates functional information, rank/threshold truncated products of p methods (Dudbridge & Koeleman, 2003; Moskvina et al., 2009; Zaykin, Zhivotovsky, Westfall, & Weir, 2002), set‐based analysis as implemented in PLINK (Chang et al., 2015; Purcell et al., 2007), Brown's method (Brown, 1975; Moskvina et al., 2011) that adjusts for the LD structure between SNPs, a logistic kernel‐machine based test that accounts for nonlinear SNP effects (Wu et al., 2010), MAGMA (de Leeuw, Mooij, Heskes, & Posthuma, 2015) that uses a regression‐based approach and Pascal (Lamparter, Marbach, Rueedi, Kutalik, & Bergmann, 2016) that utilizes the sum and maximum of chi‐squared statistics to generate a set score. Each of these methods have advantages and limitations, however, the above methods do not incorporate the effect sizes from external data with individual genotype data. Set‐based methods using individual SNP *P* values are also able to improve power by incorporating external data available from previous studies using meta‐analysis. In the present paper, we focus on methods which use individual genotype data as this is a necessary requirement of PRS.

MAGMA v1.06 (de Leeuw et al., 2015) is a recent approach that has emerged as a widely used and computationally efficient set‐based method. This regression‐based approach accounts for LD between SNPs when individual genotype data are available. The matrix of SNPs within the set is decomposed into principal components (PCs), and PCs with small eigenvalues are removed. The remaining PCs are then used as uncorrelated predictors in regression against the phenotype of interest and an *F*‐test is used to determine the strength of the association between the set and the phenotype, providing the MAGMA set‐based *P*‐value. The MAGMA programme can be used on individual genotypes using the Principal Component Analysis (PCA) method and also on summary statistics using Brown's method (Brown, 1975). It often happens that summary statistics are available from a large consortium, while in‐house studies with individual genotypes have smaller sample sizes. In such situations, the options for using MAGMA are either applying the PCA method to the in‐house genotype dataset, or to a meta‐analysis of the summary statistics for both datasets.

PRS analysis can be considered as set‐based analysis when a set includes all SNPs in the whole genome. PRSs provide a method for combining information from individual SNPs into a single measure of risk allele burden. In their most widely used form, PRS's have been applied to genome‐wide SNP data where they can capture a useful fraction of genetic liability to polygenic traits. PRS's can also be used as genome‐wide predictors of affected status (Escott‐Price et al., 2015; Purcell et al., 2009; Ripke et al., 2014). We reasoned that the basic principles of polygenic score analysis can also be applied to individual genes, or to gene‐set analyses. The motivation for doing so is somewhat different than PRS analyses of genome‐wide data; rather than predict case–control status or trait liability captured, our goal in applying the principles of PRS to genes and gene sets is to detect association to these potentially biologically informative features. As for genome‐wide analyses, genes or gene‐set PRS could be used to predict affected status, or to estimate the gene‐ or pathway‐specific SNP liability captured by GWAS. However, for polygenic disorders where risk is dispersed across hundreds of genes and multiple gene sets, self‐evidently, gene‐ or pathway‐specific SNP liability will be lower than the liability captured by genome‐wide data, and accordingly, such tests will afford less case‐control discriminatory power. Risk scores for each individual per set can be used to stratify individuals for follow‐up studies and prioritize genes for further functional studies.

In this paper, we suggest a novel approach to a set‐based framework that combines advantages of MAGMA's PCA method and PRS. The proposed POlygenic LD‐Adjusted RIsk Score (POLARIS) method aims to improve upon the standard PRS method by correcting the inflated Type I error observed both in standard PRS in the presence of LD (Chatterjee et al., 2013), and also in set‐based analyses as the number of SNPs in the set grows (de Leeuw, Neale, Heskes, & Posthuma, 2016). We use spectral decomposition of the SNP correlation matrix to adjust the individuals' allele counts for LD structure. In this paper, POLARIS is presented as a self‐contained set‐based approach in that it compares the test statistic for the set with the null hypothesis, rather than a competitive approach that accounts for the baseline level of association across the genome. However, it can be turned into a competitive method either by including a general PRS in the analysis, or comparing the set‐based PRS to those generated from random sets of genes (matched for number of SNP‐sets/set size/numbers of SNPs).

POLARIS informs the analysis with previously reported effect sizes of the SNPs' association with disease. An LD‐adjusted PRS is calculated per person per set, and the overall set effect is computed using regression. Because the score is used as a predictor in a regression analysis, it is possible to include further population covariates or any other possible confounders. POLARIS uses all available information, because all PCs are incorporated into a score, thus avoiding overfitting that may result from only including the top PCs. As in standard PRS analysis, only one independent variable (apart from extra covariates) is present in the regression model, rather than the number of predictors being equal to the number of markers, or the number of chosen PCs. Like the standard PRS approach, an advantage of our method is that it performs a self‐contained test of association in the test dataset, leveraging the discovery set to increase the power of this test. A significant test statistic implies significant association specifically in the test sample, unlike a significant meta‐analysis result, where the association evidence could result from other samples. This might be important if the test sample is of specific interest, for example, a different ethnicity, or a different, but related, phenotype.

In the present paper, POLARIS is evaluated by comparing the set‐based results calculated using LD‐adjusted PRS with those found using MAGMA (de Leeuw et al., 2015) on simulated data, both with a simple constructed LD structure and real data LD pattern.

The POLARIS set‐based analysis tool is available to download from github.com/BakerEA/POLARIS. The tool is written in Python and will operate on any computing platform.

## 2 METHODS

### 2.1 POLARIS rationale and derivation

For *M* SNPs in a set, the standard PRS combines single‐SNP genotypes $g_{i}\;\left(i=1,\cdots ,M\right)$ into a single regression predictor using single‐SNP effect sizes (log(OR_*i*_) = $\beta_{i}$) taken from a previous study as coefficients,

(1) $$PRS=\sum_{i=1}^{M}\beta_{i}g_{i}=\beta^{T}g.$$

This method implements a two‐stage approach, where independent discovery and test sets are available. The effect sizes β are determined from the discovery set and vector of the number of risk alleles *g* is obtained from the test set. The underlying assumption is that individual genotypes are available for the test set, but only summary data (effect sizes β) for the discovery set are available.

The standard PRS method does not adjust for LD between markers and thus requires LD pruning (Chatterjee et al., 2013). If markers are in LD, the simple weighted sum (Equation 1) may give them undue weight; indeed, if they are in positive LD, they are likely to have a similar single‐SNP effect size and act together, thus giving a larger contribution to the PRS than a single or uncorrelated marker.

We correct for this imbalance due to LD by replacing the vector *g* of genotypes with a vector $\tilde{g}$ of adjusted dosages. Consider the spectral decomposition of the M×M marker–marker correlation matrix *C*,

$$C=\sum_{k=1}^{M}\lambda_{k}\;x_{k}\;x_{k}^{T}$$

with eigenvalues $\lambda_{k}$ satisfying ∑k=1Mλk= tr C=M and orthonormal (column) eigenvectors $x_{k}$. The correlation matrix is the covariance matrix of the joint distribution of individual genotypes after standardization of each SNP. Its eigenvectors indicate the directions of the principal axes of this standardized distribution, and the corresponding eigenvalues give the variances of the distribution in the corresponding directions. In the absence of LD, these variances will be equal to 1, and the distribution will be isotropic. However, if there is LD, then these variances will in general be different, and the standardized distribution will be more elongated in some principal directions and flattened in others.

This anisotropy can be removed by scaling the standardized joint distribution in the direction of each principal axis with the inverse square root of the eigenvalue in this direction. However, adjusting the standardized distribution in this way will not only remove LD, but also equalize the single marker variances, thus discarding information such as the minor allele frequencies. As our aim is to adjust for LD only, but not for single‐SNP variances, we therefore have chosen to apply the same scaling transformation to the original, unstandardized joint distribution instead.

More specifically, due to the orthonormality of the eigenvectors, the PRS can be expressed in a spectral decomposition

$$PRS=\beta^{T}g=\sum_{k=1}^{M}\beta^{T}x_{k}\;x_{k}^{T}g.$$

The component $x_{k}\;x_{k}^{T}g$, which is the part of *g* along the *k*th principal axis, has correlation matrix eigenvalue $\lambda_{k}$ and therefore contributes a disproportionate amount of variance to PRS unless $\lambda_{k}\approx 1$. For an uncorrelated marker, one spectral component will be concentrated on this marker, and the corresponding eigenvalue $\lambda_{k}\approx 1$.

For our adjustment, we rescale the coordinate of *g* in the direction of the *k*th principal axis, $x_{k}^{T}g$, with the inverse square root of the correlation eigenvalue, giving an adjusted coordinate $\frac{1}{\sqrt{\lambda_{k}}}\;x_{k}^{T}g$, and hence the rescaled spectral component $\frac{1}{\sqrt{\lambda_{k}}}\;x_{k}\;x_{k}^{T}g$.

Applying this adjustment to each principal axis will result in an isotropic distribution in which the correlation has mostly been removed, for the adjusted dosage vectors

$$\tilde{g}=\sum_{k=1}^{M}\frac{1}{\sqrt{\lambda_{k}}}\;\left(x_{k}^{T}g\right)=C^{-\frac{1}{2}}g.$$

Note this adjustment of multivariate data by correlation is analogous to the calculation of the Mahalanobis distance for mean zero data *x*, $x^{T}S^{-1}x={∥S^{-\frac{1}{2}}x∥}^{2}$, where *S* is the covariance matrix (Mahalanobis, 1936, see also Hotelling, 1931), except that here we use the correlation matrix instead of the covariance matrix in order to avoid adjusting for single‐marker variance.

Using the adjusted dosages $\tilde{g}$ instead of the original genotype vectors *g*, we obtain an LD‐adjusted PRS

$$\begin{array}{ccc} \sum_{i=1}^{M}\beta_{i}\tilde{g_{i}} & = & \beta^{T}\tilde{g}=\beta^{T}C^{-\frac{1}{2}}g\\ & = & \sum_{i=1}^{M}\beta_{i}\left(\sum_{k=1}^{M}\frac{1}{\sqrt{\lambda_{k}}}\;x_{k}\left(i\right)\sum_{j=1}^{M}x_{k}\left(j\right)\;g_{j}\right). \end{array}$$

In the sum over the spectral components, indexed by *k*, the terms with $\lambda_{k}=0$, corresponding to principal directions with no variance, are to be omitted, resulting effectively in a pseudoinverse of the square root of *C*. In cases of extreme LD, where $\lambda_{k}\approx 0$, this formula will apply a large correction factor to the corresponding component, thus possibly amplifying small deviations due to, for example, genotyping error. In order to avoid this instability, we introduce a ridge parameter λ_0_, for which we suggest the choice $\lambda_{0}=\sqrt{\frac{1}{N}}$, where *N* is the number of individuals in the test data, and modify the adjustment to mitigate the effect of small $\lambda_{k}$. This gives rise to the POLARIS risk score,

(2) $$\mathrm{POLARIS}=\beta^{T}\sqrt{1+\lambda_{0}}{\left(C+\lambda_{0}I\right)}^{-\frac{1}{2}}g$$

(3) $$=\sum_{i=1}^{M}\beta_{i}\left(\sum_{k=1}^{M}\sqrt{\frac{1+\lambda_{0}}{\lambda_{k}+\lambda_{0}}}\;x_{k}\left(i\right)\sum_{j=1}^{M}x_{k}\left(j\right)g_{j}\right)=\beta^{T}\tilde{g},$$

where now $\tilde{g}=\sqrt{1+\lambda_{0}}{\left(C+\lambda_{0}I\right)}^{-\frac{1}{2}}g=\sum_{k=1}^{M}\sqrt{\frac{1+\lambda_{0}}{\lambda_{k}+\lambda_{0}}}x_{k}\;x_{k}^{T}g$, and *I* is the M×M unit matrix. Note that if all markers are uncorrelated, then $\lambda_{k}\approx 1$ for all *k*, which makes $\tilde{g}\approx g$, and consequently POLARIS≈PRS.

We remark further that we applied the adjustment $\tilde{g}=C^{-\frac{1}{2}}g$ (or the extension with a ridge parameter) directly to the vector of genotypes. More precisely, an adjustment of the variance only will be achieved by removing the sample mean vector $\hat{g}$ before the adjustment, giving

$$\tilde{g}=\hat{g}+C^{-\frac{1}{2}}\left(g-\hat{g}\right)=C^{-\frac{1}{2}}g+\left(I-C^{-\frac{1}{2}}\right)\hat{g};$$

however, this only amounts to shifting the POLARIS score by a constant $\beta^{T}\left(I-C^{-\frac{1}{2}}\right)\hat{g}$, which is irrelevant in the subsequent regression analysis.

### 2.2 POLARIS: Set‐based analysis applied to simulated data

To understand detailed differences and similarities between MAGMA (de Leeuw et al., 2015) and POLARIS, we tested both methods on simulated data, both with a simple extreme LD pattern and a real‐data LD pattern between SNPs. We tested Type I and II errors by simulating null effects and introducing some association to the SNPs, respectively. We ran a set of experiments to compare the proposed POLARIS method to MAGMA.

To generate summary statistic data and genotype data, a simulated dataset was randomly split into discovery and test sets. The summary statistics for each SNP in the discovery set were computed. The following different scenarios were simulated.

- Scenario A (“One LD Block”): 10 SNPs in an LD Block with (i) $r^{2}=0.2$ and (ii) $r^{2}=0.8$ between consecutive SNPs. The “causal” SNP is associated with disease with OR=1.1 and the remaining nine SNPs have an OR closer to the null value of 1. An additional 90 independent unassociated SNPs are also present in the set, see supplementary Figure S1 for LD structure.
- Scenario B (“Real Data LD”): 115 SNPs from real genetic and environmental risk in Alzheimer's disease (GERAD) data (Harold et al., 2009), see the next section for a detailed description of the data and supplementary Figure S2 for LD structure. For an SNP in a block of strong LD, a number of controls who were homozygous for the risk allele were set to cases, and an equal number of cases homozygous for the protective allele were set to controls, thus producing an association with disease.

For these scenarios, the sample size of the discovery dataset was varied in order to determine the influence of the discovery set sample size on the POLARIS method. For Scenario A, simulations were run creating data with *N* = 20,000 and 60,000 individuals, these were split equally to result in a test and discovery set each with *N* = 10,000 and 30,000 subjects, respectively. Additionally, the larger set with 60,000 individuals was split such that the test set had *N* = 10,000 and the discovery set had *N* = 50,000 individuals. Scenario B has 13,164 subjects for the combined discovery and test sets; we divided these data 50/50 and 25/75. In both the discovery and test datasets, 30% of the sample size were cases.

A total of 1,000 simulations were performed for each scenario. The power to detect the association between the set and disease is calculated as the proportion of *P* values from the 1,000 simulations that were below a given *P*‐value threshold; the *P*‐value thresholds used were *P* = 0.05, 0.01, and 0.001. Ten thousand simulations were used for the real data simulations, thus enabling a more stringent threshold of 0.0001 to be considered. The power of the POLARIS method, applied to the test dataset and informed by the discovery dataset, was compared to the power of MAGMA both applied to the test dataset only and to the total unsplit data samples.

### 2.3 POLARIS: Gene‐based analysis applied to real data

Both POLARIS and MAGMA were applied to genotyped AD data to determine gene‐wide *P* values. The GERAD (Harold et al., 2009) genome‐wide association study (GWAS) data (3,332 cases, 9,832 controls) were used as the test dataset. International Genomics of Alzheimer's Project (IGAP) (Lambert et al., 2013) data (17,008 cases, 37,154 controls) excluding GERAD subjects (IGAP‐noGERAD) were used as the discovery data in order to inform POLARIS with association effect sizes.

International Genomics of Alzheimer's Project (IGAP) is a large study that used genotyped and imputed data on 7,055,881 single nucleotide polymorphisms (SNPs) to meta‐analyze four previously published GWAS datasets consisting of 17,008 AD cases and 37,154 controls (The Genetic and Environmental Risk in AD consortium‐GERAD, The European Alzheimer's disease Initiative‐EADI, the Alzheimer Disease Genetics Consortium‐ADGC, and The Cohorts for Heart and Aging Research in Genomic Epidemiology consortium‐CHARGE).

For this study, we used only directly genotyped SNPs from the GERAD data (cf. Escott‐Price et al., 2014, where however, imputed genotype data were used for IGAP summary statistics analysis). The GERAD and IGAP‐noGERAD datasets have 419,048 SNPs in common. It was necessary to ensure that SNP alleles were coded in the same direction across both the discovery (IGAP‐noGERAD) and test (GERAD) datasets. If alleles in IGAP‐noGERAD were coded in the opposite direction to those in GERAD, the summary effect size for the SNP was inverted. SNPs with alleles AT, TA, CG, or GC were excluded because the direction of the effect could not always be determined when combining two studies. Of the SNPs in IGAP‐noGERAD, 103,356 matched those in GERAD, the remaining had effect sizes inverted and no SNPs were excluded due to ambiguity. An MAF filter of 0.01 was applied to the data.

The missing genotypes in real data were imputed as in PLINK (Chang et al., 2015; Purcell et al., 2007), where missing genotypes are substituted by 2×MAF for each SNP. In the GERAD data, 0.0514% of genotypes required imputation.

SNPs were assigned to genes using GENCODE (v19) gene models (Harrow et al., 2012). Only genes with known gene status and those marked as protein coding were used. No window was used around the gene, only SNPs within the start and end position of the gene were included. SNPs that belong to multiple genes were assigned to all those genes. A total of 202,504 SNPs were assigned to 14,620 distinct genes with a maximum of 1,342 SNPs in a gene.

The results of gene‐based analyses for Alzheimer's disease (AD) data using POLARIS were compared to those from the MAGMA‐PCA approach in GERAD genotype data and also the MAGMA‐SUMMARY approach in IGAP data (not MAGMA‐PCA, as the individual genotypes for the whole IGAP data were not available to us). For the latter, we only consider SNPs present in both IGAP and GERAD. Prior to the gene‐set analysis, SNP summary statistics for the whole IGAP data were adjusted for the genomic control parameter, λ=1.087, as reported in Escott‐Price et al. (2014).

## 3 RESULTS

### 3.1 Set‐based analysis: Applied to simulated data

#### 3.1.1 Type I error

We investigated the Type I error rates in simulations where none of the SNPs have an association to disease (i.e., OR = 1) in either the discovery or test sets, for Scenarios A (one LD block) and B (real data LD), termed A(null) and B(null), respectively. Type I error is deemed acceptable if the nominal value is included in the 95% CI for estimated Type I error rate. The expected Type I error is displayed on the Type I error plots (gray dashed line).

**Figure 1:**
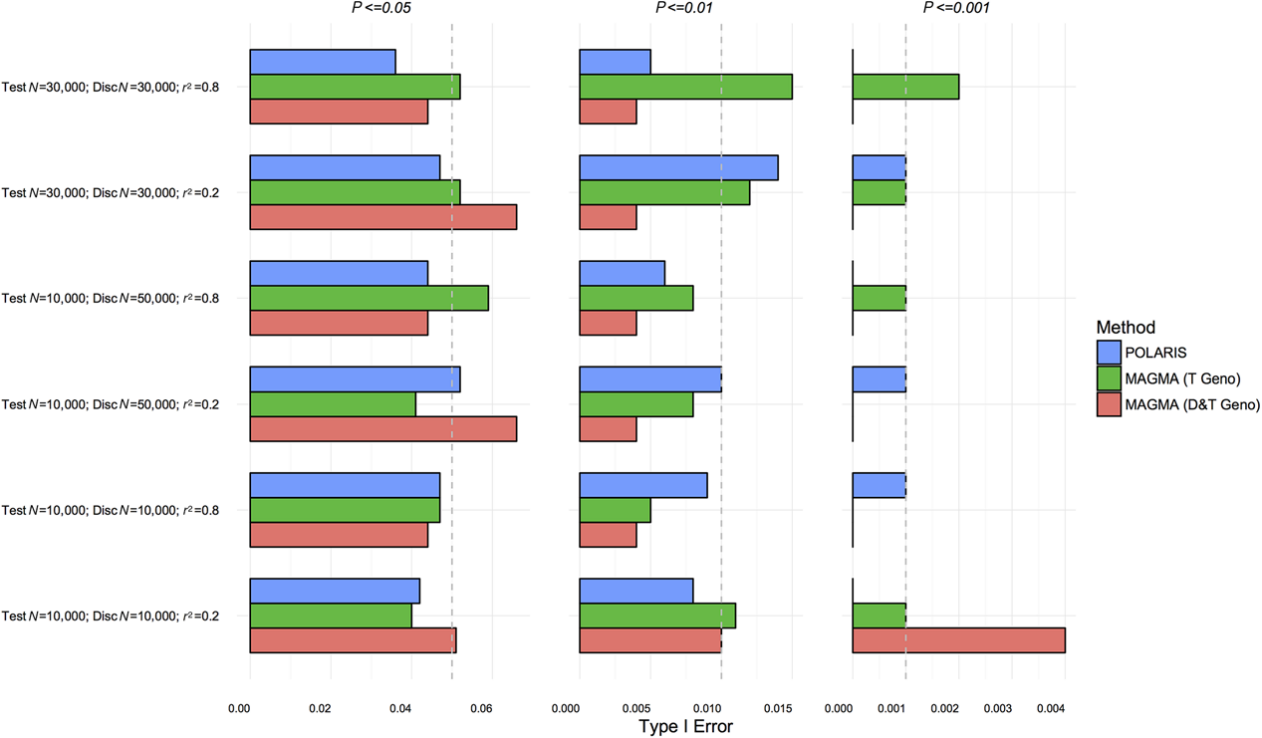
Type I error comparison of set‐based methods at different *P*‐value thresholds; scenario A(null)—simulation of 10 SNPs in LD and 90 independent SNPs *Notes*: POLARIS with no associated SNPs in both test and discovery sets (blue), MAGMA in the test set only (green), and MAGMA in combined test and discovery sets (red). Expected Type I error is shown by the gray dashed line.

Figure 1 shows Scenario A(null). The LD structure for Scenario A can be seen in supplementary Figure S1. Type I error for POLARIS is shown by the blue bars (POLARIS), the red bars (MAGMA D&T Geno) display the Type I error for the MAGMA method in the combined discovery and test individual genotype data and green bars (MAGMA T Geno) show the Type I error for MAGMA in test set genotype data only. In supplementary Figure S3, we additionally show the Type I error rate for summary statistics based analysis using MAGMA (MAGMA D&T Summ).

The Type I error rate is reasonable in the majority of cases; the nominal value is included in the 95% CI. MAGMA in the combined data has slightly inflated Type I error at a *P*‐value threshold of 0.001 when the test and discovery set have *N* = 10000 and $r^{2}=0.2$.

**Figure 2:**
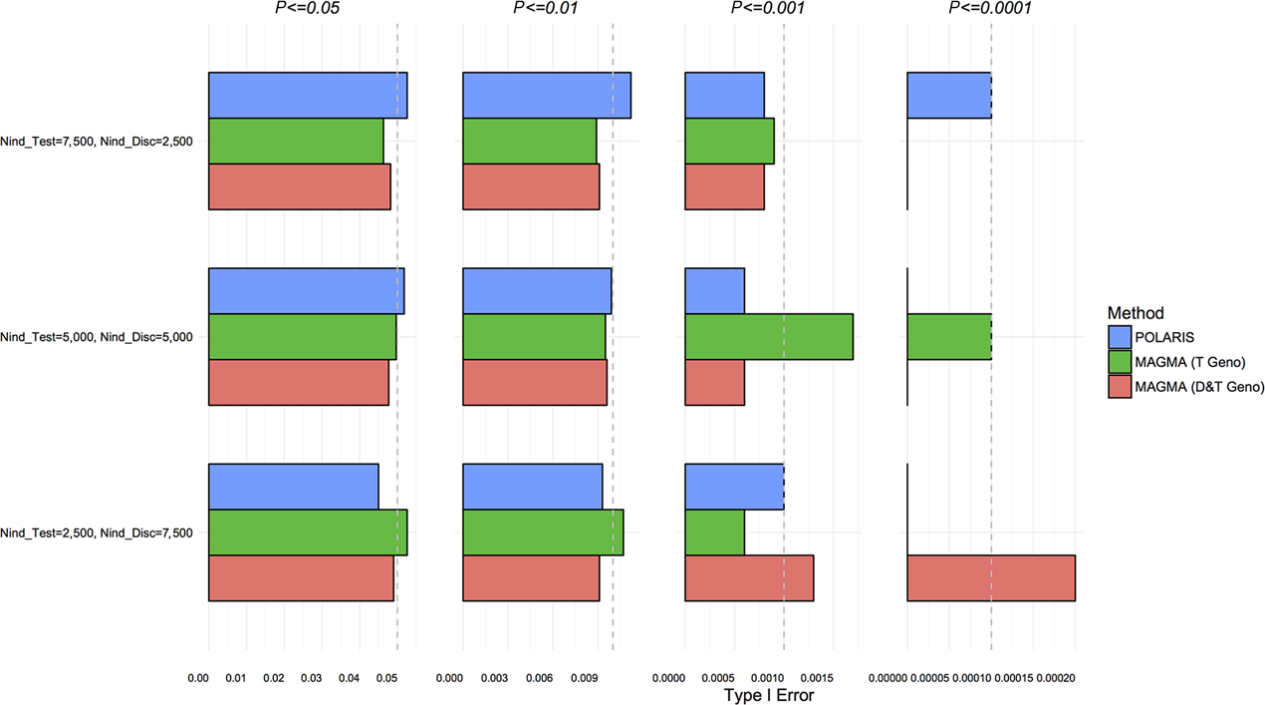
Type I error comparison of set‐based methods; scenario B(null)—simulation of 115 SNPs from real data, with permuted phenotypes to remove effect sizes *Notes*: POLARIS with no associated SNPs in either test or discovery sets (blue), MAGMA in test set only (green), and MAGMA in combined test and discovery sets (red) are compared. Expected Type I error is shown by the gray dashed line.

The Type I error for scenario B(null), with the case‐control status randomly permuted in order to remove the effect size of any SNPs, is shown in Figure 2 and supplementary Figure S4. The LD structure for this scenario can be observed in supplementary Figure S2. The Type I error rate is within 95% CIs of expected values for most cases, but is somewhat inflated for the summary statistic based analysis using MAGMA.

#### 3.1.2 Power

**Figure 3:**
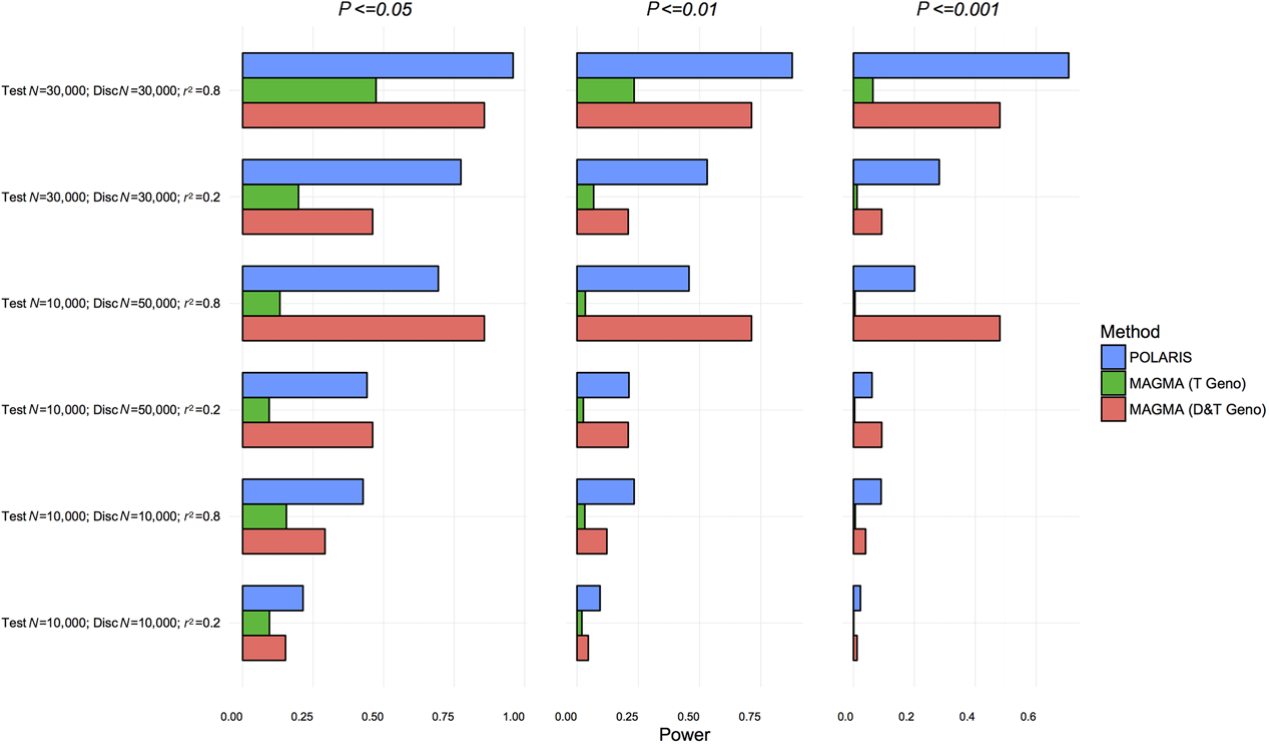
Power comparison of set‐based methods at different *P*‐value thresholds; scenario A—simulation of 10 SNPs in LD with OR=1.1 and 90 independent, unassociated SNPs *Notes*: POLARIS (blue), MAGMA in the test set only (green), and MAGMA in combined test and discovery sets (red) are compared.

The power of the POLARIS method (blue bars, POLARIS), MAGMA in the test set only (green bars, MAGMA T Geno) and MAGMA in the combined discovery and test sets (red bars, MAGMA D&T Geno) are displayed for each simulated scenario. The power graphs for Scenario A are shown in Figure 3. The 10 SNPs that are in LD are associated with disease with OR = 1.1.

POLARIS has equivalent power compared with MAGMA in the combined discovery and test sets in all cases. In the most likely realistic situation, the discovery set is larger than the test set, but only summary statistics are available for the discovery set. MAGMA on the combined dataset has higher power where the test *N* = 10,000 and discovery *N* = 50,000, but here MAGMA is applied to the individual genotypes of the discovery and test sets combined (*N* = 60,000), so the sample used to estimate LD and perform the statistical test is very large, whereas POLARIS uses the discovery set *N* = 50,000 for effect size estimation and only *N* = 10,000 for LD estimation, and importantly, for statistical testing. In all cases, POLARIS has higher power than MAGMA in the test set only, as is expected, because POLARIS increases power by incorporating information from the discovery set. The power for POLARIS increases when the size of the test set increases, as this improves the estimate of LD between markers.

**Figure 4:**
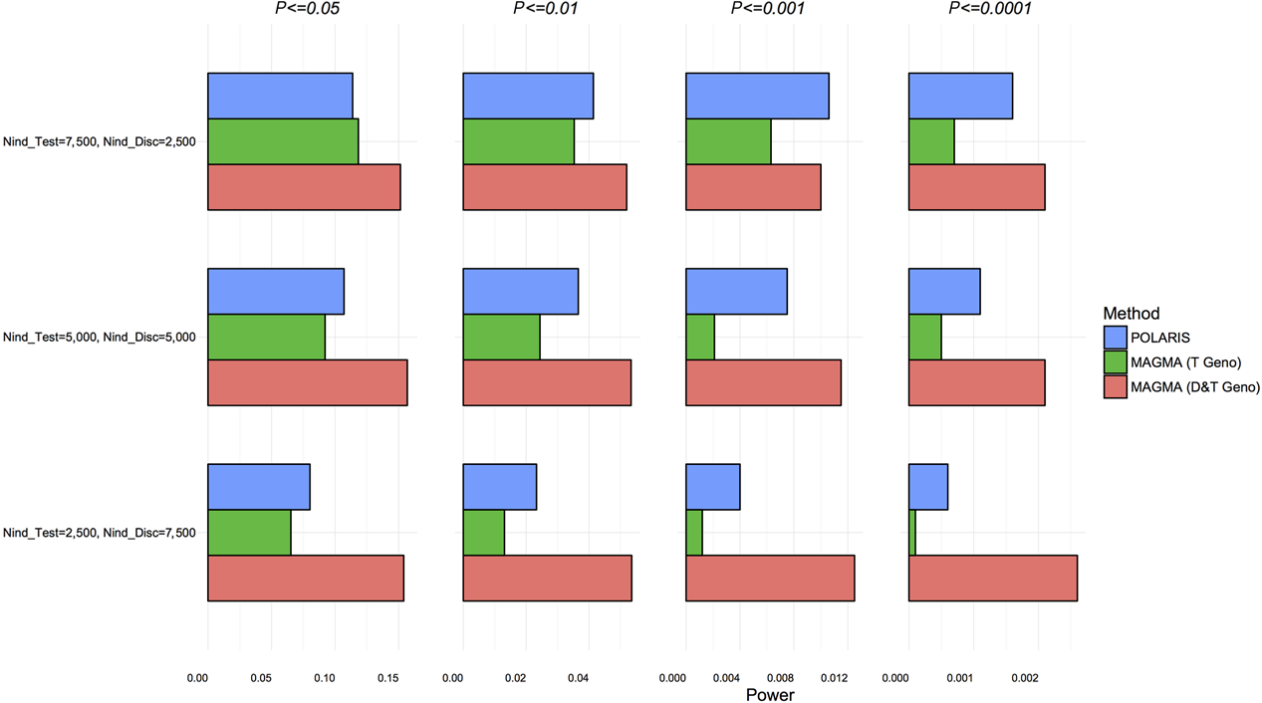
Power comparison of set‐based methods; scenario B—simulation of 115 SNPs, with a proportion of phenotypes permuted to maintain effect sizes *Notes*: POLARIS (blue), MAGMA in the test set only (green) and MAGMA in combined test and discovery sets (red) are compared.

Figure 4 shows the power graph for Scenario B. The power of the POLARIS method lies generally between the power of MAGMA applied to the test set only and MAGMA in the combined test and discovery sets. One can see that by using the information from the discovery set, POLARIS increases the power compared to using the test set only, but, as is to be expected, not as much as using the individual genotypes from the discovery set as well as the test set.

These power results were also compared with the power of the summary statistics based approach implemented in MAGMA, see supplementary Figures S5 and S6. Note that the power of the summary statistics based approach (MAGMA D&T Summ) exceeds the power of MAGMA PCA (MAGMA D&T Geno) on the same combined dataset.

### 3.2 Gene‐based analysis: Application to real data

Table 1 demonstrates the number and proportion of genes below a particular *P*‐value threshold for POLARIS, MAGMA‐PCA in GERAD genotype data and MAGMA‐SUMMARY in IGAP summary statistic data. Statistically independent associations in some instances implicate overlapping regions. To define genes as physically independent, we have annealed associated genes that were not separated by at least 250 kb in each analysis separately. In the APOE region, significant genes on chromosome 19 between 44.4 and 46.5 Mb were counted as one. We also present the results for all genes in supplementary Table S1.

**Table 1:** Comparison of the number and proportion of independent genes below a *P*‐value threshold for POLARIS, MAGMA‐PCA in GERAD data and MAGMA‐SUMMARY in IGAP data

	POLARIS		MAGMA‐PCA in GERAD		MAGMA‐SUMMARY in IGAP	
P‐value threshold	Number of genes	Proportion of genes	Number of genes	Proportion of genes	number of genes	proportion of genes
1*	563		581		560	
0.05	302	0.5364	283	0.4871	255	0.4554
0.01	116	0.2060	98	0.1687	114	0.2036
0.001	19	0.0337	12	0.0207	31	0.0554
0.0001	7	0.0124	4	0.0069	12	0.0214
0.00001	3	0.0053	2	0.0034	9	0.0161
0.000001	2	0.0036	1	0.0017	5	0.0089

*Note that the total number of genes (*P*‐value threshold equal to 1) differs, this is due to some gene exclusions made by MAGMA software.

The number of independent significant genes for all *P*‐value thresholds is higher or equal for POLARIS compared to the MAGMA‐PCA approach in GERAD data. This is expected as POLARIS uses both GERAD and IGAP‐noGERAD data, while MAGMA‐PCA uses GERAD genotypes only. The results for the summary statistic approach show higher numbers of significant genes for higher significance thresholds. The five gene‐wide significant genes found by the summary statistics approach are: *TOMM40*, *CLU*, *BIN1*, *MS4A4E*, and *CR1*, which have all been previously reported as being associated with AD from single SNP analyses (Harold et al., 2009 and Lambert et al., 2013). For these five genes, POLARIS also finds an association, but does not always reach gene‐wide significance ($P=6.33\times 10^{-24}$, $P=7.17\times 10^{-6}$, 0.00112, 0.00108, and 0.00065, respectively).

The issue of bias in the estimation of set‐based *P* values caused by gene size is insufficiently tackled by most available methods (de Leeuw et al., 2016; Ruderfer, 2013). Larger genes harbor a larger number of SNPs, and if each SNP has a small inflation in *P*‐value due to, for example, unaccounted stratification, then these large genes will show greater accumulated inflation. To assess whether this is an issue in POLARIS, the phenotypes in GERAD data were permuted to create 1,000 simulations, and for each gene, the empirical *P*‐value (the proportion of *P* values less than 0.05) was computed. The correlation between the number of SNPs per gene and the empirical *P* value of each gene in AD data was then determined. We found no evidence (*r* = 0.0009, *P* = 0.9096) of a correlation between the number of SNPs in a gene and the gene *P*‐value for the POLARIS method. Therefore, associations with disease observed in larger genes is not simply due to a greater number of SNPs in the gene. Similarly, we observed no evidence (*r* = –0.00321, *P* = 0.6977) of an inflation in *P*‐value for increasing gene size using MAGMA‐PCA on GERAD data. When considering the correlation between the IGAP gene‐based *P*‐value and set size, we observe a statistically significant negative correlation (*r* = –0.083, *P* $<2.2\times 10^{-16}$) when MAGMA‐SUMMARY is used on summary data, indicating that the higher the number of SNPs, the lower the set‐based *P*‐value.

## 4 DISCUSSION

In this paper, we present a method for accounting for LD in the calculation of a PRS. The resulting individual LD‐adjusted PRS can also be used for analyzing whether a set of SNPs is associated with disease. This method combines the advantages of PRS and spectral analysis of the genetic data. The latter suggests a mathematically sound adjustment for LD and includes a stabilization parameter (similar to ridge regression) to cope with cases of extreme LD. It adjusts for LD between SNPs and informs the analysis with previously reported effect sizes of a SNP's association with disease. In the present study, we have chosen to do this adjustment using the SNP‐SNP correlation matrix; however, one could alternatively use the SNP‐SNP covariance matrix. For all examples above, this gives very similar results. Partitioning the overall polygenic risk based on meaningful SNP sets, the method allows both to test for significance of association of these sets (set‐based analysis) and to provide individual set‐specific risk scores for subjects, which can further be used for risk prediction of subphenotypes with respect to the SNP sets. To assess the quality of the proposed approach, we compare its use for set‐based analysis with the widely used MAGMA software. We show that POLARIS gives the correct Type I error and its power lies between that of MAGMA applied to the test dataset only and MAGMA applied to the combined test and discovery datasets. In practice, researchers would use all the available genotype data, and would use PRS‐based methods only if effect sizes only are known for an additional dataset.

POLARIS has four main advantages. (1) It produces a risk score per person per set, unlike other set‐based methods which only provide a *P*‐value for the strength of association between the set and disease. This set risk score can be used to stratify individuals for follow up studies (e.g., clinical trials) and also prioritize genes for further functional studies (e.g., animal models), supporting the development of precision medicines. (2) POLARIS can increase power by leveraging the discovery set to perform a self‐contained test of association in the test dataset. Another way to incorporate the discovery set would be to use meta‐analysis, however, this detects an association in the combined set rather than the test set only. This may be important when the test data differs in some way from the discovery data, for example different ethnicity, or different phenotype. A good example might be where the test sample uses different diagnostic criteria to measure the same phenotype (e.g., self‐report questionnaire for depression) and one wishes to validate these criteria by showing that they show association to the same genes as those implicated by the standard diagnosis. (3) POLARIS is not inflated by set size. This is an issue previously reported in summary statistic based approaches (Chatterjee et al., 2013; Ruderfer, 2013) and is shown to be the case using MAGMA on the IGAP summary statistic data. (4) The overall set association can easily be adjusted by population or any other covariates.

For set‐based analyses, in situations where only summary statistics are available for part of the data, we suggest to use the POLARIS method which unites advantages of the PRS approach and the PCA‐based set‐based method, while taking into account the LD structure for the genetic region of interest.

POLARIS can be used for any set of SNPs, for example, the whole genome, genes, or pathways. Therefore, it has the potential of data‐driven discovery of pathways.

POLARIS can also be utilized in a number of cross‐disorder analyses to determine commonality between disorders at a gene‐based or pathway‐based level. There are a number of common disorders for which the GWAS summary data are publically available (e.g., Psychiatric Genomics Consortium). The GWAS data for one disorder can be used to generate scores per person per gene in another disorder or subphenotype of interest, and thus test for overlap between disorders at a gene‐based level.

The POLARIS method can be extended to add additional information into the score, such as rare variants from exome sequencing studies. The POLARIS set‐based method is implemented into a freely accessible platform independent software. Large sets have a high computational burden due to the spectral decomposition of large correlation matrices; recommendations on maximum set size and the corresponding required computational resource are included with the software.

In this study, POLARIS was applied to test binary traits. However, the POLARIS score can also be used as a variable (along with other covariates) in regression models for quantitative traits.

A limitation of the POLARIS implementation is that currently it is only available as a self‐contained set‐based method. However, POLARIS can in principle be used as a competitive set analysis, adjusting for the baseline level of association in the data either by including a general PRS in the analysis or comparing the set‐based PRS to those generated from random sets of genes (matched for number of genes/gene size/numbers of SNPs) or random sets of SNPs (matched for LD, MAF, and SNP density).

Another limitation of PRS‐type approaches is the imperfect tagging of the underlying causal variants by SNPs and imperfect effect size estimates. The challenge of selecting the true set of susceptibility SNPs for PRS modeling to capture heritability has been pointed out (Chatterjee, Shi, & Garcia‐Closas, 2016). Our approach can use all SNPs in a set of interest, even when in LD, and therefore any causal genotyped SNPs will be included. If the causal SNPs are not present in the sample, then the tagging SNPs only are used. The effect sizes of all SNPs in LD will be adjusted according to the LD structure, not according to the causal/noncausal nature of the SNP.

POLARIS is a valuable extension to standard PRS by adjusting for LD between markers and removing the necessity to LD prune data prior to analysis. POLARIS provides a test of the set's association with disease while also producing subject specific risk scores.

## Supporting information

Figure S1: The LD Structure of the 100 SNPs used in Scenario A – Simulation of 10 SNPs in LD with OR=1.1 and 90 independent, unassociated SNPs.

Figure S2: The LD Structure of the 115 SNPs used in Scenario B – Simulation of 115 SNPs from Real Data, with a Proportion of Phenotypes Permuted to Maintain Effect Sizes.

Figure S3: Type I Error Comparison of Set‐Based Methods at Different P‐value Thresholds; Scenario A(null)‐ Simulation of 10 SNPs in LD and 90 independent SNPs. POLARIS (purple), MAGMA in the test set only (blue), MAGMA in combined test and discovery sets (green) and MAGMA using the combined test and discovery set summary statistics (red). Expected Type I Error is shown by the grey dashed line.

Figure S4: Type I Error Comparison of Set‐Based Methods; Scenario B(null) ‐ Simulation of 115 SNPs from Real Data, with Permuted Phenotypes to Remove Effect Sizes. POLARIS (purple), MAGMA in the test set only (blue), MAGMA in combined test and discovery sets (green) and MAGMA using the combined test and discovery set summary statistics (red) are compared. Expected Type I Error is shown by the grey dashed line.

Figure S5: Power Comparison of Set‐Based Methods at Different P‐value Thresholds; Scenario ASimulation of 10 SNPs in LD with OR=1.1 and 90 independent, unassociated SNPs. POLARIS (purple), MAGMA in the test set only (blue), MAGMA in combined test and discovery sets (green) and MAGMA using the combined test and discovery set summary statistics (red) are compared.

Figure S6: Power Comparison of Set‐Based Methods; Scenario B – Simulation of 115 SNPs, with a Proportion of Phenotypes Permuted to Maintain Effect Sizes. POLARIS (purple), MAGMA in the test set only (blue), MAGMA in combined test and discovery sets (green) and MAGMA using the combined test and discovery set summary statistics (red) are compared.

**Table SI: Comparison of the Number and Proportion of Independent Genes Below a P‐value Threshold for POLARIS, MAGMA‐PCA in GERAD data and MAGMA‐SUMMARY in IGAP data**.

## APPENDIX 1

**Genetic and Environmental Risk for Alzheimer's Disease Consortium (GERAD1) Collaborators** Authors who contributed to the generation of original study data for GERAD but not to the current publication and their affiliations are included herein.

Denise Harold^1^, Richard Abraham^1^, Paul Hollingworth^1^, Rebecca Sims^1^, Amy Gerrish^1^, Jade Chapman^1^, Giancarlo Russo^1^, Marian Hamshere^1^, Jaspreet Singh Pahwa^1^, Valentina Escott‐Price^1^, Nandini Badarinarayan^1^, Kimberley Dowzell^1^, Amy Williams^1^, Nicola Jones^1^, Charlene Thomas^1^, Alexandra Stretton^1^, Angharad Morgan^1^, Sarah Taylor^1^, Simon Lovestone^2^, John Powell^3^, Petroula Proitsi^3^, Michelle K Lupton^3^, Carol Brayne^4^, David C. Rubinsztein^5^, Michael Gill^6^, Brian Lawlor^6^, Aoibhinn Lynch^6^, Kevin Morgan^7^, Kristelle Brown^7^, Peter Passmore^8^, David Craig^8^, Bernadette McGuinness^8^, Stephen Todd^8^, Janet Johnston^8^, Clive Holmes^9^, David Mann^10^, A. David Smith^11^, Seth Love^12^, Patrick G. Kehoe^12^, John Hardy^13^, Simon Mead^14^, Nick Fox^15^, Martin Rossor^15^, John Collinge^14^, Wolfgang Maier^16^, Frank Jessen^16^, Reiner Heun^16^, Britta Schürmann^16, 17^, Alfredo Ramirez^16^, Tim Becker^18^, Christine Herold^18^, André Lacour^18^, Dmitriy Drichel^18^, Hendrik van den Bussche^19^, Isabella Heuser^20^, Johannes Kornhuber^21^, Jens Wiltfang^22^, Martin Dichgans^23, 24^, Lutz Frölich^25^, Harald Hampel^26^, Michael Hüll^27^, Dan Rujescu^28^, Alison Goate^29^, John S.K. Kauwe^30^, Carlos Cruchaga^31^, Petra Nowotny^31^, John C. Morris^31^, Kevin Mayo^31^, Gill Livingston^32^, Nicholas J. Bass^32^, Hugh Gurling^32^, Andrew McQuillin^32^, Rhian Gwilliam^33^, Panagiotis Deloukas^33^, Ammar Al‐Chalabi^34^, Christopher E. Shaw^34^, Andrew B. Singleton^18^, Rita Guerreiro^18, 35^, Thomas W. Mühleisen^36, 37^, Markus M. Nöthen^36, 37^, Susanne Moebus^38^, Karl‐Heinz Jöckel^38^, Norman Klopp^39^, H‐Erich Wichmann^39, 40, 41^, Minerva M. Carrasquillo^42^, V. Shane Pankratz^43^, Steven G. Younkin^42^, Peter Holmans^1^, Michael O'Donovan^1^, Michael J.Owen^1^, Julie Williams^1^.

^1^Medical Research Council (MRC) Centre for Neuropsychiatric Genetics and Genomics, Neurosciences and Mental Health Research Institute, Department of Psychological Medicine and Neurology, School of Medicine, Cardiff University, Cardiff, United Kingdom. ^2^Department of Psychiatry, Medical Sciences Division, University of Oxford, Oxford, United Kingdom. ^3^Kings College London, Institute of Psychiatry, Department of Neuroscience, De Crespigny Park, Denmark Hill, London, United Kingdom. ^4^Institute of Public Health, University of Cambridge, Cambridge, United Kingdom. ^5^Cambridge Institute for Medical Research, University of Cambridge, Cambridge, United Kingdom. ^6^Mercers Institute for Research on Aging, St. James Hospital and Trinity College, Dublin, Ireland. ^7^Institute of Genetics, Queens Medical Centre, University of Nottingham, NG7 2UH, United Kingdom. ^8^Ageing Group, Centre for Public Health, School of Medicine, Dentistry and Biomedical Sciences, Queens University Belfast, United Kingdom. ^9^Division of Clinical Neurosciences, School of Medicine, University of Southampton, Southampton, United Kingdom. ^10^Clinical Neuroscience Research Group, Greater Manchester Neurosciences Centre, University of Manchester, Salford, United Kingdom. ^11^Oxford Project to Investigate Memory and Ageing (OPTIMA), University of Oxford, Department of Pharmacology, Mansfield Road, Oxford OX3 9DU, United Kingdom. ^12^University of Bristol Institute of Clinical Neurosciences, School of Clinical Sciences, Frenchay Hospital, Bristol, United Kingdom. ^13^Department of Molecular Neuroscience and Reta Lilla Weston Laboratories, Institute of Neurology, UCL, London, United Kingdom. ^14^MRC Prion Unit, Department of Neurodegenerative Disease, UCL Institute of Neurology, London, United Kingdom. ^15^Dementia Research Centre, Department of Neurodegenerative Diseases, University College London, Institute of Neurology, London, United Kingdom. ^16^Department of Psychiatry, University of Bonn, Sigmund‐Freud‐Straβe 25, 53105 Bonn, Germany. ^17^Institute for Molecular Psychiatry, University of Bonn, Bonn, Germany ^18^Laboratory of Neurogenetics, National Institute on Aging, National Institutes of Health, Bethesda, MD, 20892, United States. ^19^Institute of Primary Medical Care, University Medical Center Hamburg‐Eppendorf, Germany. ^20^Department of Psychiatry, Charité Berlin, Germany. ^21^Department of Psychiatry, Friedrich‐Alexander‐University Erlangen‐Nürnberg, Germany. ^22^Department of Psychiatry and Psychotherapy, University Medical Center (UMG), Georg‐August‐University, Göttingen, Germany. ^23^Institute for Stroke and Dementia Research, Klinikum der Universität München, Marchioninistr. 15, 81377 Munich, Germany. ^24^Department of Neurology, Klinikum der Universität München, Marchioninistr. 15, 81377 Munich, Germany. ^25^Central Institute of Mental Health, Medical Faculty Mannheim, University of Heidelberg, Heidelberg, Germany. ^26^Institute for Memory and Alzheimer's Disease & INSERM, Sorbonne Universities, Pierre and Marie Curie University, Paris, France; Institute for Brain and Spinal Cord Disorders (ICM), Department of Neurology, Hospital of Pitié‐Salpétrière, Paris, France. ^27^Centre for Geriatric Medicine and Section of Gerontopsychiatry and Neuropsychology, University of Freiburg, Freiburg, Germany. ^28^Department of Psychiatry, University of Halle, Halle, Germany. ^29^Institute for Molecular Psychiatry, University of Bonn, Bonn, Germany. ^30^Department of Biology, Brigham Young University, Provo, Utah 84602, United States of America. ^31^Departments of Psychiatry, Neurology and Genetics, Washington University School of Medicine, St Louis, Missouri 63110, United States of America. ^32^Department of Mental Health Sciences, University College, London, United Kingdom. ^33^The Wellcome Trust Sanger Institute, Wellcome Trust Genome Campus, Hinxton, Cambridge, United Kingdom. ^34^MRC Centre for Neurodegeneration Research, Department of Clinical Neuroscience, Institute of Psychiatry, Kings College London, London, SE5 8AF, United Kingdom. ^35^Department of Molecular Neuroscience, Institute of Neurology, University College London, Queen Square, London WC1N 3BG, United Kingdom. ^36^Department of Genomics, Life & Brain Center, University of Bonn, Sigmund‐Freud‐Str. 25, D‐53127 Bonn, Germany. ^37^Institute of Human Genetics, University of Bonn, Wilhelmstr. 31, D‐53111 Bonn, Germany. ^38^Institute for Medical Informatics, Biometry and Epidemiology, University Hospital of Essen, University Duisburg‐Essen, Hufelandstr. 55, D‐45147 Essen, Germany. ^39^Institute of Epidemiology, Helmholtz Zentrum München, German Research Center for Environmental Health, 85764 Neuherberg, Germany. ^40^Institute of Medical Informatics, Biometry and Epidemiology, Chair of Epidemiology, Ludwig‐Maximilians‐Universität, Munich, Germany. ^41^Klinikum Grosshadern, Munich, Germany. ^42^Department of Neuroscience, Mayo Clinic College of Medicine, Jacksonville, Florida 32224, United States of America. ^43^Division of Biomedical Statistics and Informatics, Mayo Clinic and Mayo Foundation, Rochester, Minnesota 55905, United States of America.
