# Supplementary figures and images for: POLARIS: Polygenic LD‐adjusted risk score approach for set‐based analysis of GWAS data
Source: Genet Epidemiol. 2018 Mar 12;42(4):366–77. doi: 10.1002/gepi.22117 (PMC6001515; doi:10.1002/gepi.22117)

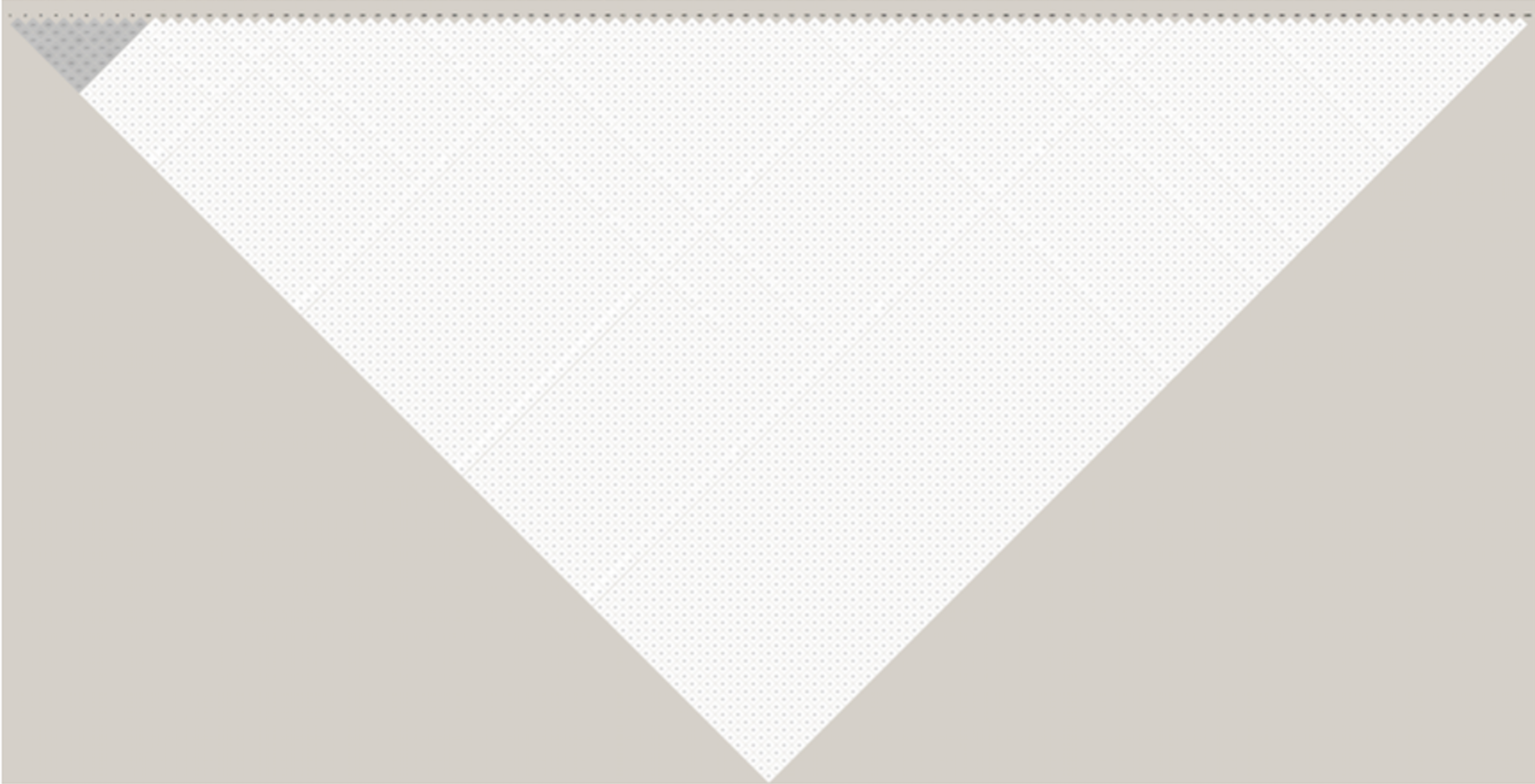

Supplement: Supplementary file 1 — Figure S1: The LD Structure of the 100 SNPs used in Scenario A – Simulation of 10 SNPs in LD with OR=1.1 and 90 independent, unassociated SNPs. [file GEPI-42-366-s001.tiff]

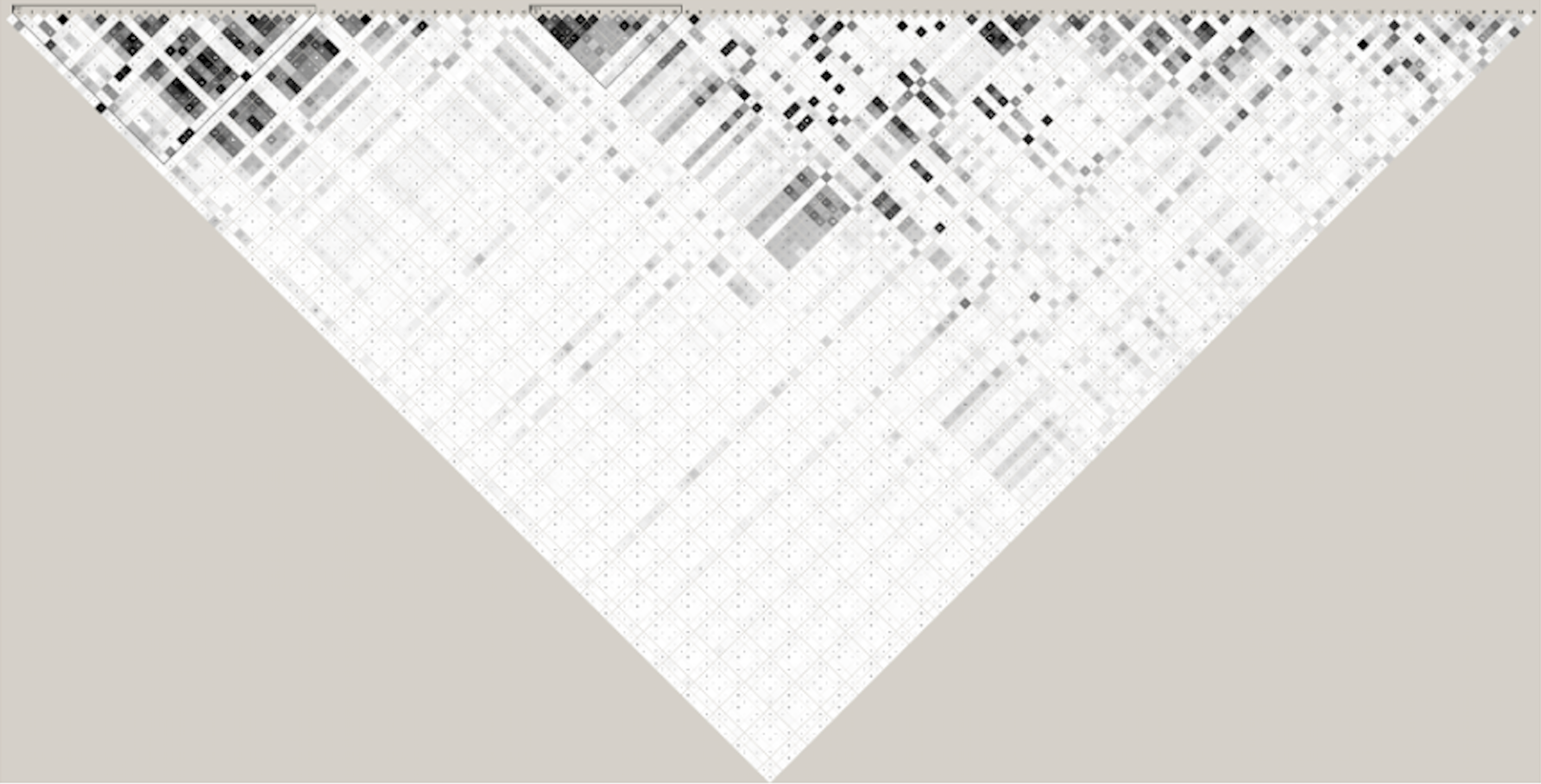

Supplement: Supplementary file 2 — Figure S2: The LD Structure of the 115 SNPs used in Scenario B – Simulation of 115 SNPs from Real Data, with a Proportion of Phenotypes Permuted to Maintain Effect Sizes. [file GEPI-42-366-s002.tiff]

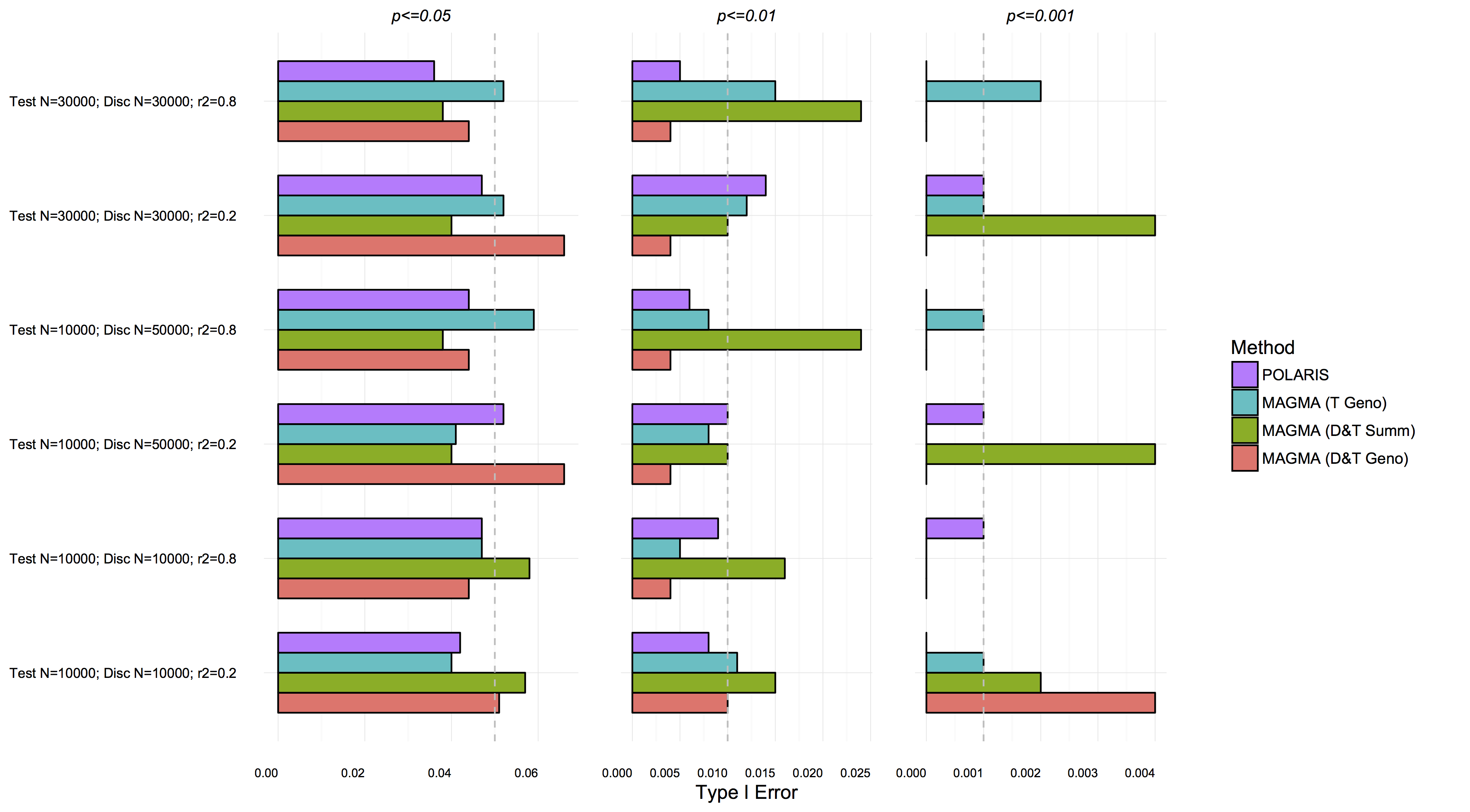

Supplement: Supplementary file 3 — Figure S3: Type I Error Comparison of Set‐Based Methods at Different P‐value Thresholds; Scenario A(null)‐ Simulation of 10 SNPs in LD and 90 independent SNPs. POLARIS (purple), MAGMA in the test set only (blue), MAGMA in combined test and discovery sets (green) and MAGMA using the combined test and discovery set summary statistics (red). Expected Type I Error is shown by the grey dashed line. [file GEPI-42-366-s003.tiff]

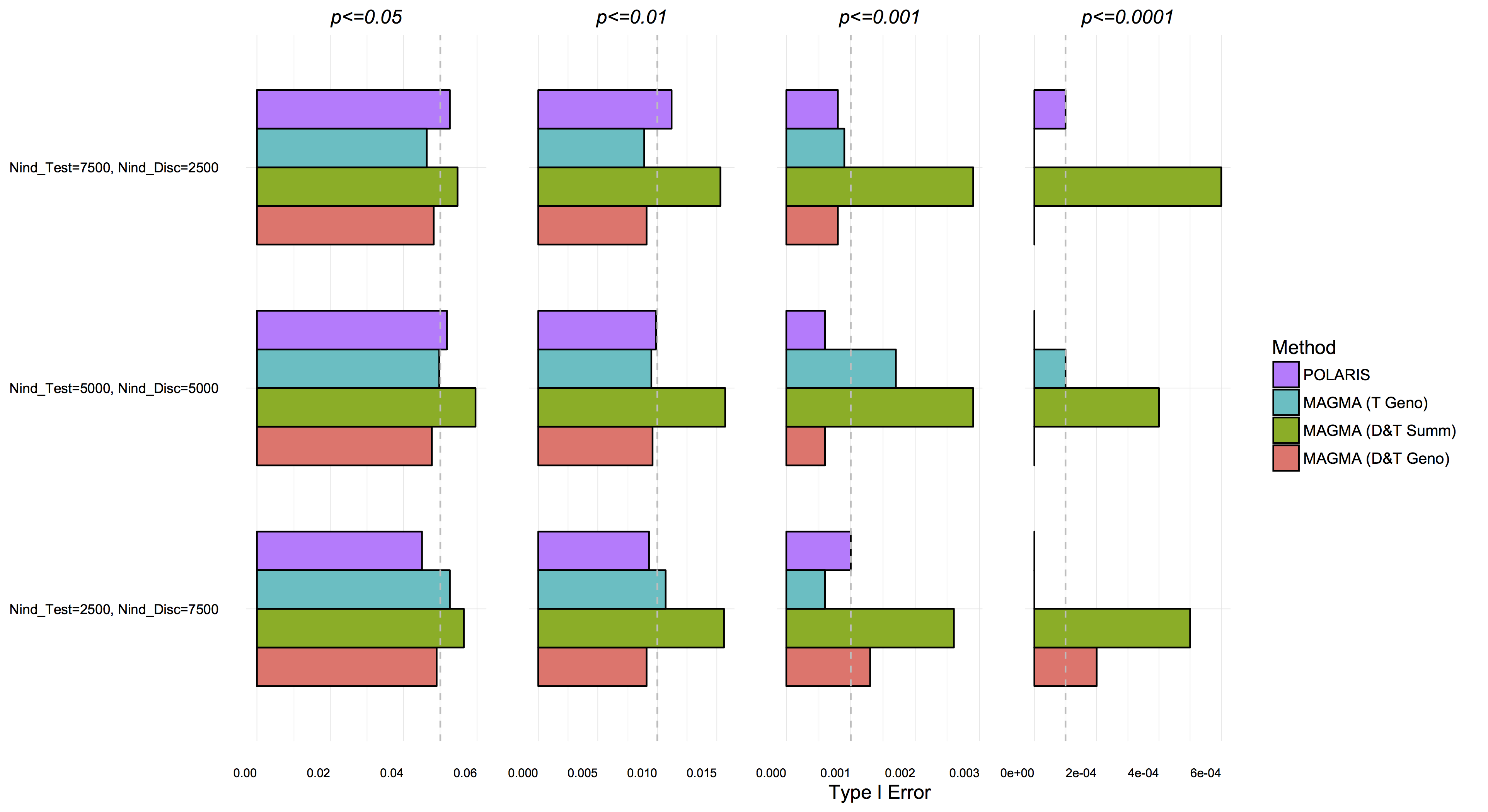

Supplement: Supplementary file 4 — Figure S4: Type I Error Comparison of Set‐Based Methods; Scenario B(null) ‐ Simulation of 115 SNPs from Real Data, with Permuted Phenotypes to Remove Effect Sizes. POLARIS (purple), MAGMA in the test set only (blue), MAGMA in combined test and discovery sets (green) and MAGMA using the combined test and discovery set summary statistics (red) are compared. Expected Type I Error is shown by the grey dashed line. [file GEPI-42-366-s004.tiff]

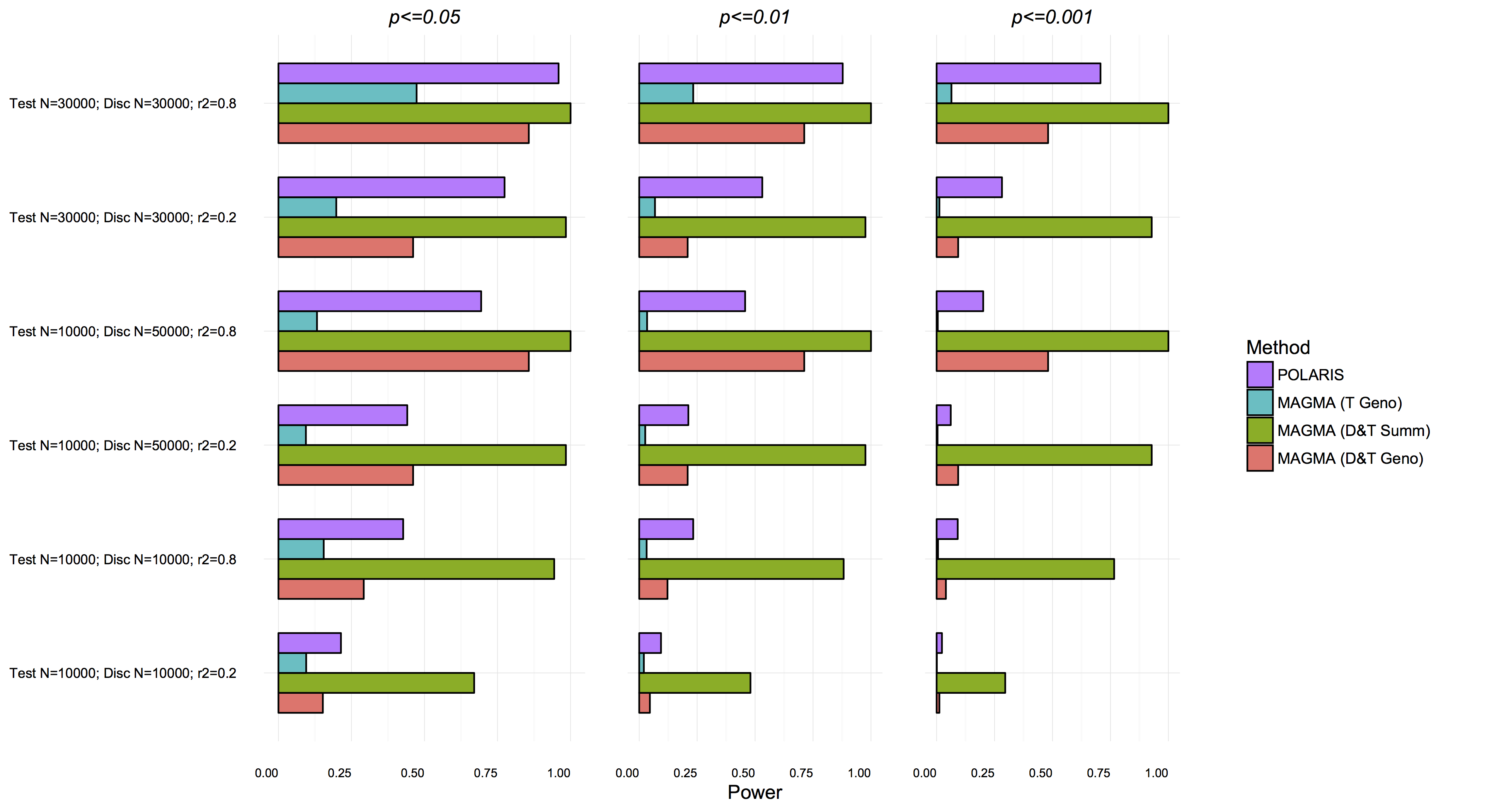

Supplement: Supplementary file 5 — Figure S5: Power Comparison of Set‐Based Methods at Different P‐value Thresholds; Scenario ASimulation of 10 SNPs in LD with OR=1.1 and 90 independent, unassociated SNPs. POLARIS (purple), MAGMA in the test set only (blue), MAGMA in combined test and discovery sets (green) and MAGMA using the combined test and discovery set summary statistics (red) are compared. [file GEPI-42-366-s005.tiff]

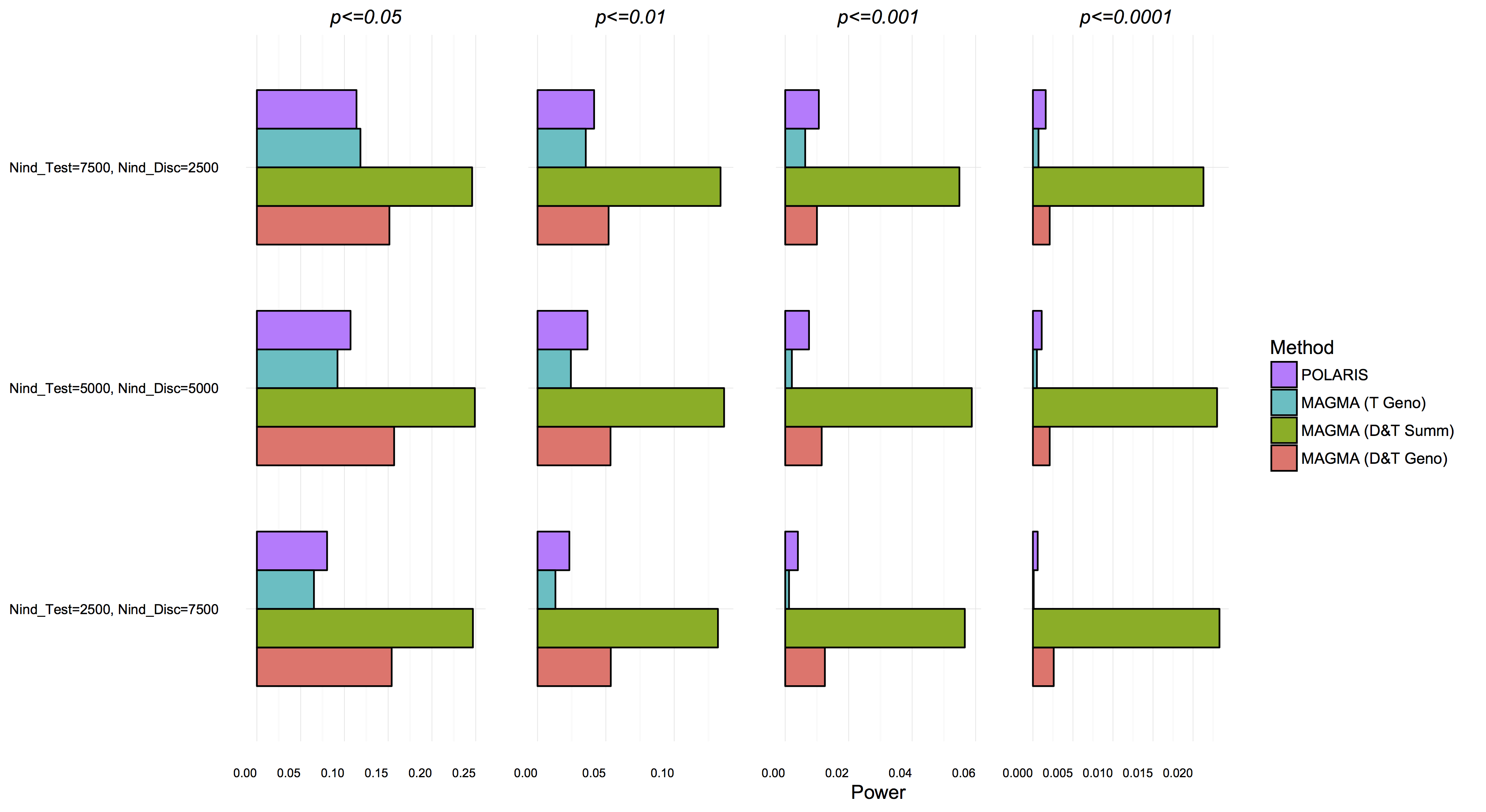

Supplement: Supplementary file 6 — Figure S6: Power Comparison of Set‐Based Methods; Scenario B – Simulation of 115 SNPs, with a Proportion of Phenotypes Permuted to Maintain Effect Sizes. POLARIS (purple), MAGMA in the test set only (blue), MAGMA in combined test and discovery sets (green) and MAGMA using the combined test and discovery set summary statistics (red) are compared. [file GEPI-42-366-s006.tiff]
