# Supplementary material for: POLARIS: Polygenic LD‐adjusted risk score approach for set‐based analysis of GWAS data
Source: Genet Epidemiol. 2018 Mar 12;42(4):366–77. doi: 10.1002/gepi.22117 (PMC6001515; doi:10.1002/gepi.22117)
Supplement: Supplementary file 7 — Table SI: Comparison of the Number and Proportion of Independent Genes Below a P‐value Threshold for POLARIS, MAGMA‐PCA in GERAD data and MAGMA‐SUMMARY in IGAP data. [file GEPI-42-366-s007.docx]

Table SI: Comparison of the Number and Proportion of Independent Genes Below a P-value Threshold for POLARIS, MAGMA-PCA in GERAD data and MAGMA-SUMMARY in IGAP data

| P-value Threshold | POLARIS | | MAGMA-PCA in GERAD | | MAGMA-SUMMARY in IGAP | |
| --- | --- | --- | --- | --- | --- | --- |
|  | Number of Genes | Proportion of Genes | Number of Genes | Proportion of Genes | Number of Genes | Proportion of Genes |
| 1* | 14620 |  | 14606 |  | 14607 |  |
| 0.05 | 840 | 0.0575 | 749 | 0.0513 | 783 | 0.0536 |
| 0.01 | 192 | 0.0131 | 162 | 0.0111 | 244 | 0.0167 |
| 0.001 | 30 | 0.0021 | 24 | 0.0016 | 62 | 0.0042 |
| 0.0001 | 13 | 0.0009 | 9 | 0.0006 | 31 | 0.0021 |
| 0.00001 | 6 | 0.0004 | 4 | 0.0003 | 21 | 0.0014 |
| 0.000001 | 4 | 0.0003 | 3 | 0.0002 | 15 | 0.0010 |

- Note that the total number of genes (p-value threshold equal to 1) differs, this is due to some gene exclusions made by MAGMA software.
